# A Panel of Four-lncRNA Signature as a Potential Biomarker for Predicting Survival in Clear Cell Renal Cell Carcinoma

**DOI:** 10.7150/jca.40421

**Published:** 2020-04-27

**Authors:** Haoran Liu, Tao Ye, Xiaoqi Yang, Peng Lv, Xiaoliang Wu, Hui Zhou, Jin Zeng, Kun Tang, Zhangqun Ye

**Affiliations:** 1 Department of Urology, Tongji Hospital, Tongji Medical College, Huazhong University of Science and Technology, Wuhan 430030, China; 2 Hubei Institute of Urology, Wuhan 430030, China

**Keywords:** lncRNAs, Prognosis, Survival, Clear Cell Renal Cell Carcinoma

## Abstract

Long non-coding RNAs (lncRNAs) have been considered as biomarkers for the carcinogenesis and development of various cancers. However, the prognostic significance of lncRNAs in renal cell carcinoma (RCC) remains unclear. This study aimed to determine the predictive ability of lncRNAs in clear cell RCC (ccRCC). Among the cohort of kidney renal clear cell carcinoma (KIRC) of the The Cancer Genome Atlas (TCGA), 525 patients were enrolled in our study. Expression of lncRNAs based on RNAseq was obtained from TCGA. Kaplan-Meier prognostic analysis and a Cox proportional hazards regression model were used to assess related factors. The lncRNA signature was then validated in an independent cohort of an additional 60 ccRCC patients. Hierarchical clustering of the KIRC TCGA dataset identified 26 differentially expressed lncRNAs (11 down-regulated and 15 up-regulated) using average linkage clustering. Kaplan-Meier survival analysis identified 30 statistically significant lncRNAs that strongly predicted prognosis, with 4 ccRCC-specific lncRNAs (TCL6, PVT1, MIR155HG, and HAR1B) being differentially expressed and correlating significantly with OS. Patients assigned to the high-risk group were associated with poor OS compared with patients in the low-risk group (HR = 2.57; 95%CI, 1.89-3.50; p < 0.001). This finding was validated in the Tongji Hospital cohort, and the four-lncRNA signature was shown to be significantly predictive of ccRCC prognosis (p < 0.001). In this study, we constructed an applicable four-lncRNA-based classifier as a reliable prognostic and predictive tool for OS in patients with ccRCC.

## Introduction

Currently, renal cell carcinoma (RCC) has become the most general malignant tumor of the kidneys in adults, corresponding to 3.7% of all adult cancers worldwide. RCC is also an important cause of cancer-related morbidity and mortality globally 1. Clear cell renal cell carcinoma (ccRCC) is the most universal subtype, and it is very important to demonstrate the molecular changes linked to malignant transformation and longer survival 2. The current pathological grade system and tumor node metastasis (TNM) stage of the American Joint Committee on Cancer (AJCC) exhibits valuable but insufficient prediction of prognosis and estimation for subsets of RCC patients 3. Overall, clinicopathological risk factors limit their clinical application, without a clear prediction of disease recurrence, chemotherapy response or survival. As an increasing amount of evidence has demonstrated that the discovery and application of molecular biomarkers contributes to prognostic evaluation and identification of potential high-risk RCC patients 4, there is an increasing need to add new prognostic and predictive biomarkers to complement and improve the staging system currently in use. Such markers may also serve as therapeutic targets.

Long noncoding RNAs (lncRNAs), a class of noncoding RNAs, are identified as non-protein-coding transcripts of more than 200 nucleotides 5. Since our previous study on the expression patterns of genome-wide lncRNAs in RCC based on microarray, increasing evidence has demonstrated that by acting as tumor suppressors or onco-lncRNAs, aberrant expression of lncRNAs plays a vital role in the development and evolution of many types of human carcinomas. Indeed, these molecules are important in regulating multiple and complex biological processes, for instance, cell proliferation, metabolism, differentiation, angiogenesis and the epithelial-mesenchymal transition (EMT) 6. Recently, many studies have explored the value of lncRNAs as minimally invasive biomarkers for diagnosis, prognosis or monitoring curative effects in various cancers, including ccRCC 7-10. Nonetheless, more potential and valuable lncRNA biomarkers are needed to improve the clinical outcomes of ccRCC patients.

Our present study was designed to demonstrate the prognostic potential of lncRNAs in ccRCC and identify a potential panel of four-lncRNA signatures as a composite biomarker for risk stratification of ccRCC patients to complement traditional clinicopathological prognostic factors. The signature developed can help stratify ccRCC patients for optimal treatment strategies.

## Materials and methods

### Expression profiles and sample information

We downloaded the RNAseq data from TCGA, mainly containing the lncRNA dataset (Level 3) and clinical data for RCC patients based on kidney renal clear cell carcinoma (KIRC) samples using the Illumina HiSeq 2000 platform. Our study was in accord with TCGA publication guidelines. In total, 525 primary ccRCC tumor samples with detailed lncRNA expression data were collected from the current TCGA database, and the process applied met with the parameters identified in the initial-phase study 11. The patients met the following criteria: fully characterized tumors, complete OS data and RNAseq information and no pretreatment. We collected the clinicopathological characteristics of the included patients with RCC, mainly including age, gender, tumor size, laterality, TNM, tumor grade, tumor stage, and overall survival. The primary end-point in our study was OS. Extended demographic parameters of the patients, as characterized by TCGA consortium, are presented in Table S1 of Additional file 2. Because the data we collected were retrieved from TCGA, the conventional additional approval by an ethics committee was not necessary. Data processing was conducted according to TCGA human subject protection and data access policies.

For our Tongji Hospital cohort, 60 pairs of RCC and adjacent nontumor tissues were collected from patients who underwent radical nephrectomy in our hospital from December 2015 to January 2019. Approval of the Ethical Committee of Tongji Hospital was obtained, and all patients included were fully informed of our study. All of the tissue samples were collected during surgery and preserved at -80°C. Two experienced pathologists confirmed the pathological subtypes. Every sample was instantly frozen in liquid nitrogen and preserved at -80°C for later RNA extraction and qPCR analysis. All clinicopathological characteristics were obtained from electronic records, mainly including age, gender, tumor size, tumor position, tumor stage, TNM, and tumor grade. Follow-up was conducted quarterly by telephone or in the clinic. Significant survival events, including tumor progression, recurrence, metastasis and death, were recorded. Samples were obtained under informed consent and approval of the Ethics Committees of Tongji Hospital.

### Cluster analysis of datasets

Hierarchical cluster analysis was employed to explore relationships among the results of individual studies. The overall rank matrix was established on the basis of rank matrices acquired from independent analysis of up-regulated and down-regulated lncRNA profiles. Within the matrix, lncRNAs with a value of 0.5 are not reported in this study. LncRNAs with a value greater than 0.5 were considered to be up-regulated (one minus normalized rank of lncRNA from the analysis of lncRNA lists), and those values less than 0.5 were considered to be down-regulated (normalized rank as the outcome of analysis for down-regulated lncRNA lists). Spearman rank correlation combined with average linkage method was utilized in the cluster analysis.

### RNA extraction and qPCR

Total RNA was extracted from the frozen samples using TRIzol (Invitrogen) according to the manufacturer's protocol. Oligo-dT primers and superscript II reverse transcriptase (Invitrogen) were utilized to generate first-strand complementary DNA (cDNA). After that, a quantification trial of four deregulated lncRNAs was conducted by qPCR using SYBR Premix ExTaq with an MX3000. U6 primers were acquired from GeneCopoeia. qPCR was conducted under the following protocol: 95°C for 10 min, followed by 40 cycles of 95°C for 10 sec, 60°C for 20 sec and 72°C for 30 sec, with 95°C for 1 min and 60°C for 1 min. Additionally, every trials were accomplished in triplicate. Expression levels were standardized to GAPDH. The relative fold-changes of lncRNA expression were calculated using the ^ΔΔ^CT method, and the outcomes were expressed as 2 ^-ΔΔ^CT. The lncRNA primers used for qPCR validation in this study are listed in Table S3 of Additional file 2.

### Statistical analysis

OS was defined as from the day of diagnosis to the day of death or final follow-up. Patients with no events or still alive until the day of the final follow-up were censored. Statistical analysis was performed by SPSS 17.0 software, and survival curves were drawn using the Kaplan-Meier method with log-rank tests to evaluate differences between groups. LncRNAs shown to be differentially expressed (FC>1.66&FC<0.60) were further studied. Univariate Cox regression analysis was employed to estimate correlations between the overall survival of ccRCC patients and expression level of each lncRNA. Hazard ratios (HR) and 95% confidence intervals (CI) were obtained. A total of four lncRNAs were identified and separated into two parts, a high-risk group and a low-risk group, using the median of the discovery series as the cut-off point. A risk score formula to predict OS was generated on the basis of a linear combination of the expression level multiplied by the regression coefficient obtained from the Univariate Cox regression model (β): risk score = explncRNA1*βlncRNA1 + explncRNA2*βlncRNA2 + … explncRNAn*βlncRNAn. By utilizing the median risk score as the cut-off, the included ccRCC patients were separated into high-score and low-score groups. A four-lncRNA expression signature was then constructed using a linear combination of the expression levels of the four lncRNAs and the estimated regression confidence interval in the multivariate Cox regression analysis, as previously described. Kaplan-Meier survival curves with the log-rank test were used to evaluate differences in OS between the two groups with high-risk and low-risk lncRNAs. Univariate and multivariate Cox proportional hazards analyses of lncRNA expression and ccRCC patient OS in TCGA were also analyzed. A two-sided p value less than 0.05 was identified as statistically significant.

## Results

### ccRCC patient features in TCGA and the Tongji Hospital validation cohort

A total of 525 ccRCC patients with a median age of 61 from TCGA were enrolled for analysis in our study. Additionally, 60 ccRCC patients with a median age of 58.6 from the Tongji Hospital cohort were included. The clinical characteristics of the two ccRCC cohorts, including gender, tumor size, TNM stage, tumor grade, laterality, lymph node status and metastasis, are shown in Table S1 of Additional file 2. The median follow-up time was 79.5 months and 52.5 months for the cohorts from TCGA and Tongji, respectively.

### Screening of differentially expressed lncRNAs in ccRCC patients

**Figure 1:**
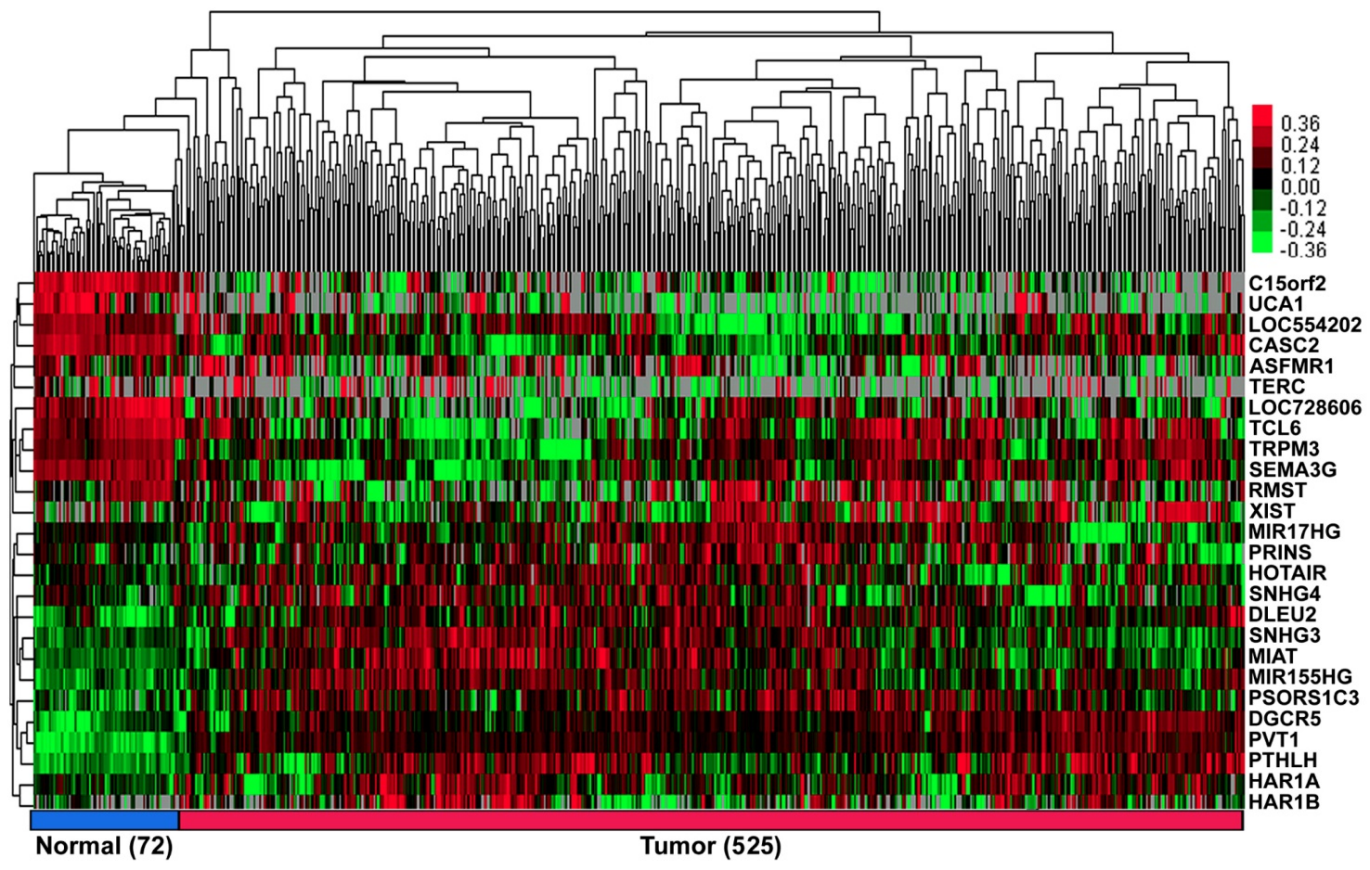
** The heat map shows the relative fold change of lncRNAs in ccRCC compared with normal adjacent tissues.** Hierarchical clustering of the KIRC TCGA dataset with 26 differentially expressed lncRNAs (11 down-regulated and 15 up-regulated) by average linkage clustering. Each row represents a single lncRNA, and each column represents a single sample. Pseudocolours show transcript levels from low to high on a log 2 scale from -3 to 3, ranging from a low (dark, black) to a high (bright, red, or green) association. Short red and green vertical bars indicate upregulated and downregulated lncRNAs, respectively. The black bar with the pseudocolour 0 indicates no signal in the RNAseq data.

According to our exclusion criterion, lncRNAs that were differentially expressed (FC>1.66&FC<0.60) were further studied. A total of 26 lncRNAs (11 downregulated, UCA1; C15orf2; LOC728606; TCL6; LOC554202; TERC; TRPM3; ASFMR1; RMST; SEMA3G and CASC2, and 15 upregulated, PVT1; PTHLH; PSORS1C3; HAR1B; DGCR5; MIR155HG; XIST; MIAT; HAR1A; SNHG4; SNHG3; PRINS; HOTAIR; DLEU2 and MIR17HG; Figure 1, Table S2 of Additional file 2) were differentially expressed in the ccRCC patients.

### Screening of lncRNAs significantly associated with overall survival in ccRCC patients

**Figure 2:**
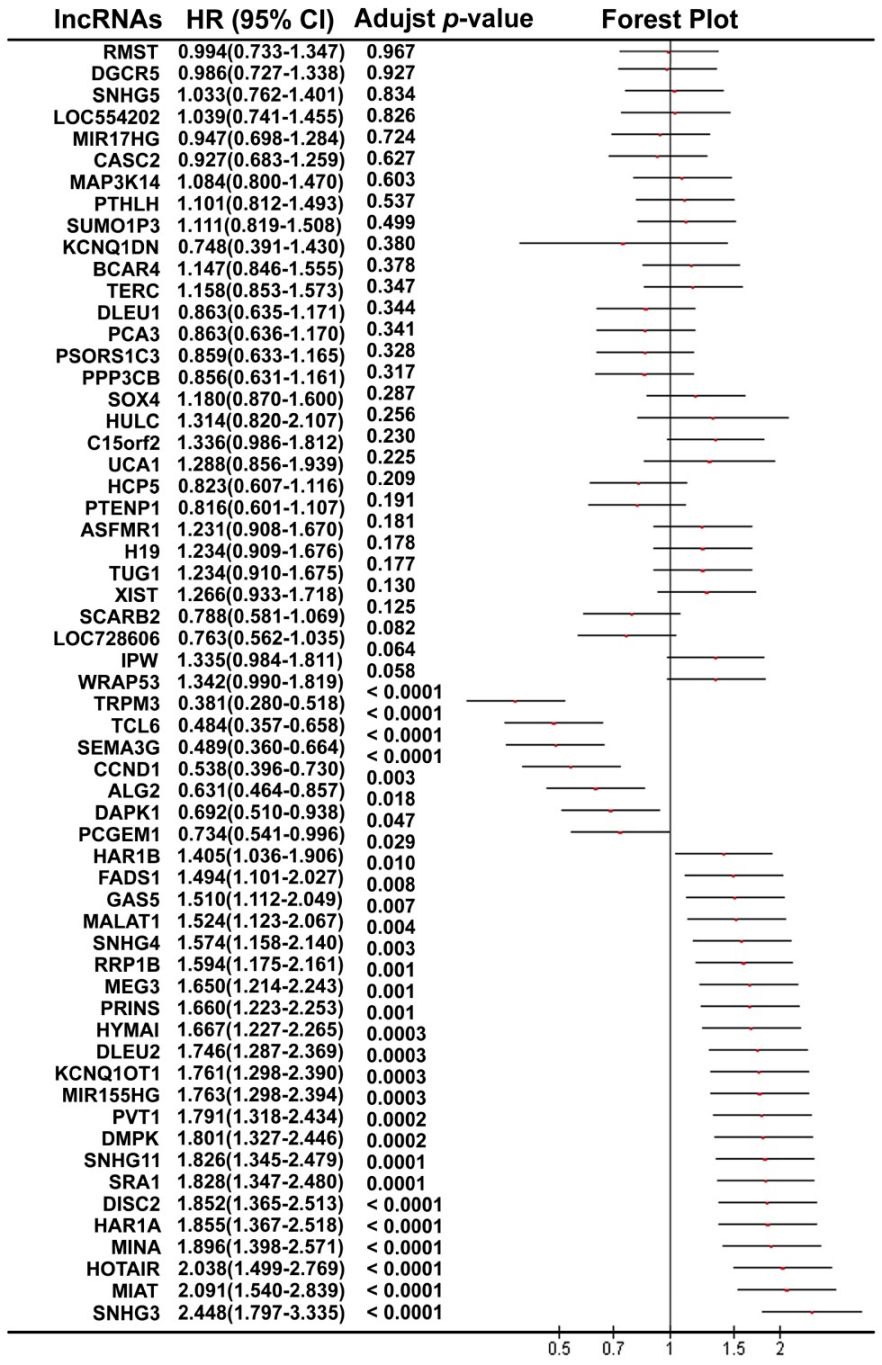
The forest plot shows the association between each lncRNA and OS in ccRCC patients using TCGA.

Using the median value of lncRNA expression as a cut-off point, we further performed the Kaplan-Meier overall survival analysis for each lncRNA in ccRCC patients using the dataset from TCGA (Figure 2). A total of 30 lncRNAs (7 protective lncRNAs, WRAP53, TRPM3, TCL6; SEMA3G, CCND1 and DAPK1; and 23 potentially risk lncRNAs, SNHG3, MIAT, HOTAIR, MINA, HAR1A, DISC2, SRA1, SNHG11, DMPK, PVT1; MIR155HG, KCNQ1OT1, DLEU2, HYMAI, PRINS, MEG3, RRP1B, SNHG4, MALAT1 GAS5, FADS1, HAR1B and PCGEM1; Figure S1 of Additional file 1) were validated to be significantly related to overall survival in the ccRCC patients.

### Identification of the four-lncRNA signature

**Figure 3:**
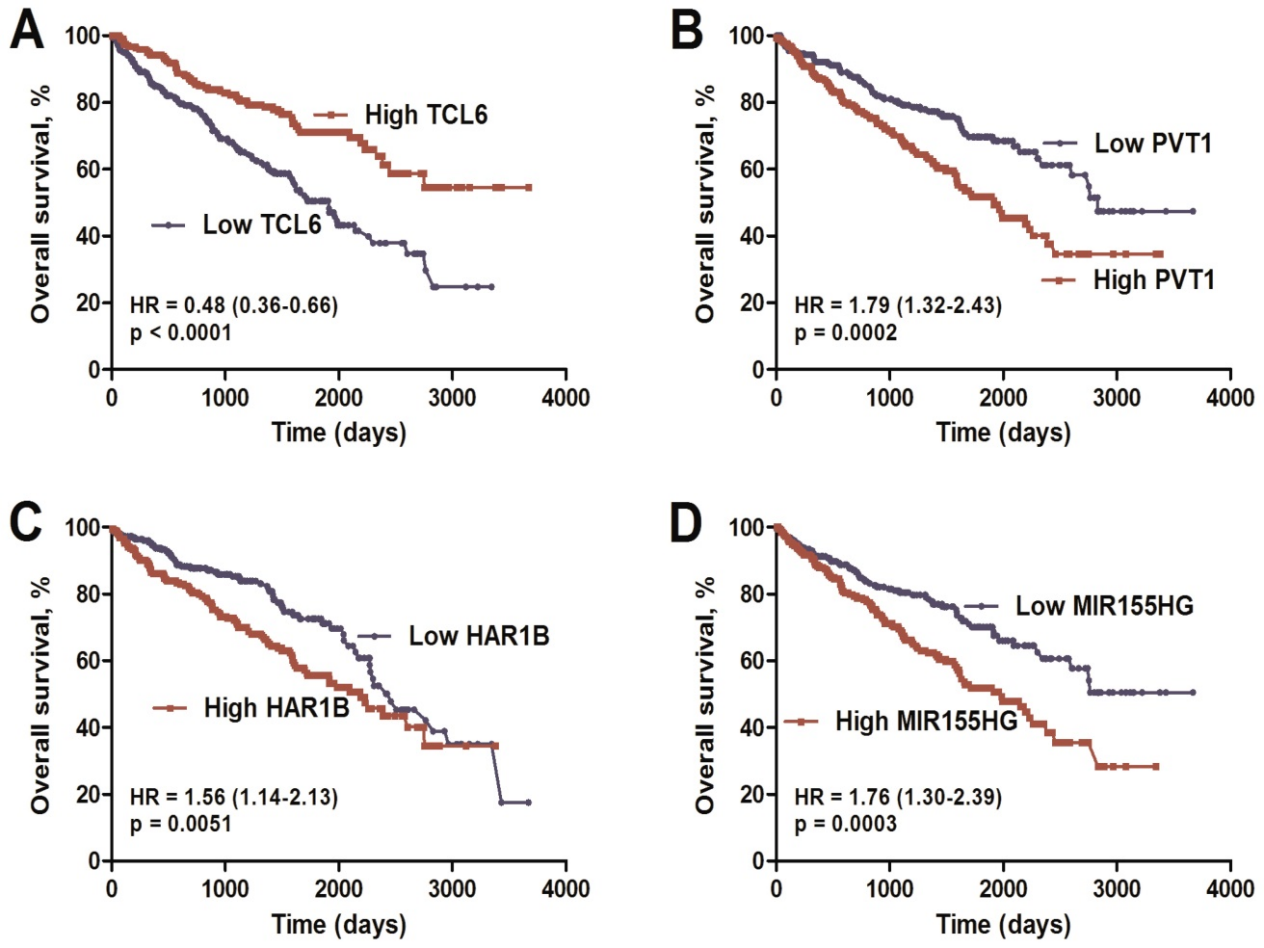
** Kaplan-Meier overall survival analysis of the four lncRNAs using the KIRC TCGA dataset.** Kaplan-Meier plots of overall survival in the KIRC TCGA cohort are shown according to lncRNA expression (TCL6 (A), PVT1 (B), MIR155HG (C), HAR1B (D)). The relative median expression value of each lncRNA was used as the cut-off point.

Combined analysis of ccRCC-specific lncRNAs, which were both differentially expressed (Figure 2; FC>1.66&FC<0.60) and significantly associated with overall survival (Table S2), was performed. Finally, we identified a four-lncRNA signature including one downregulated (TCL6) and three upregulated (PVT1, MIR155HG, and HAR1B) lncRNAs using the dataset from TCGA (Table S4 of Additional file 2). As based on Cox proportional hazards analysis, low expression of TCL6 (HR: 0.48, 95%CI: 0.36-0.66) with high expression of PVT1 (HR: 1.79, 95%CI: 1.32-2.43), MIR155HG (HR: 1.76, 95%CI: 1.30-2.39) and HAR1B (HR: 1.56, 95%CI: 1.14-2.13) was associated with poor overall survival (Figure 3).

### The lncRNA signature risk score as an independent indicator for ccRCC prognosis

**Figure 4:**
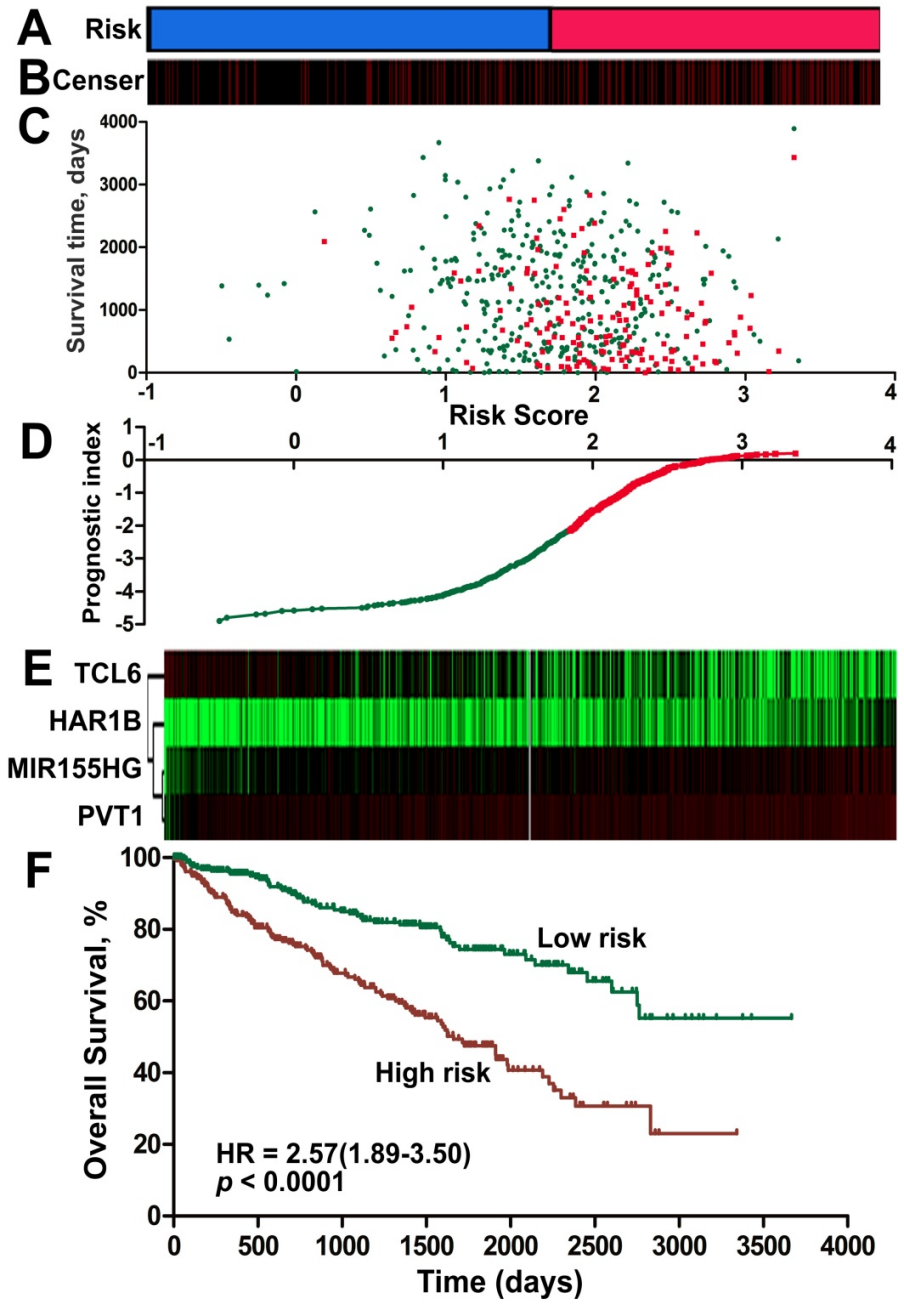
** Risk score for the 4-lncRNA signature and outcome in ccRCC patients.** Risk score of the lncRNA signature divided into low- and high-score groups. (B) Information related to the censoring event was analyzed. Columns represent ccRCC patients. The black dotted line represents the censored status, and the red line represents patient survival status. (C) Survival status and duration of cases. (D) The lncRNA signature risk score distribution. (E) Heat map of the lncRNA expression profiles. Each row represents a single lncRNA, and each column represents the corresponding patients. The black dotted line represents the median lncRNA risk score cut-off dividing patients into low-risk and high-risk groups. (F) Kaplan-Meier curve for the low-score and high-score group.

A risk-score formula was created based on the expression of these four lncRNAs for OS prediction, as follows: Risk score = (0.167*expression level of PVT1) + (0.149*expression level of MIR155HG) + (0.162* expression level of HAR1B)- (0.109*expression level of TCL6). The risk score based on the four lncRNAs was calculated for each ccRCC patient. By applying the median as the cut-off, 525 ccRCC patients were classified into a high-score group or a low-score group. The protective lncRNAs exhibited high expression in the low-score group, whereas the risk lncRNAs showed low expression in the high-score group (Figure 4, Figure S2 of Additional file 1). The ccRCC patients in the high-score group experienced a significantly worse OS (HR: 2.57, 95%CI: 1.89-3.50, p < 0.001) than those in the low-score group (Figure 4F). When we further performed Kaplan-Meier overall survival analysis of subgroups according to tumor grade (Figure S3 of Additional file 1) and stage (Figure S4 of Additional file 1) using the KIRC dataset from TCGA, the lncRNA signature risk score remained as a significant predictor for ccRCC overall survival.

Moreover, univariate Cox regression analyses showed that age (p < 0.001), laterality (p = 0.011), tumor size (p < 0.001), tumor TNM stage (p < 0.001), lymph node positivity (p = 0.024), metastasis (p < 0.001) and risk score (p < 0.001) were significantly related to the overall survival of the ccRCC patients; multivariate Cox regression analysis revealed that age (p < 0.001), tumor stage (p < 0.001) and risk score (p < 0.001) were independent prognostic factors (Table 1).

### The lncRNA signature is only specific for ccRCC

Although we constructed a promising 4-lncRNA panel for ccRCC prognosis, it is uncertain if this panel is only specific for ccRCC. Thus, additional studies were performed to further examine the changes in expression of these four lncRNAs using kidney renal papillary cell carcinoma (KIRP) and kidney chromophobe (KICH) databases. According to Kaplan-Meier curve analysis, the 4-lncRNA signature could not predict overall survival in KIRP and KICH (Figure S5 of Additional file 1).

### Confirmation of the expression and prognostic value of the top four dysregulated lncRNAs in Tongji cohort ccRCC patients

**Figure 5:**
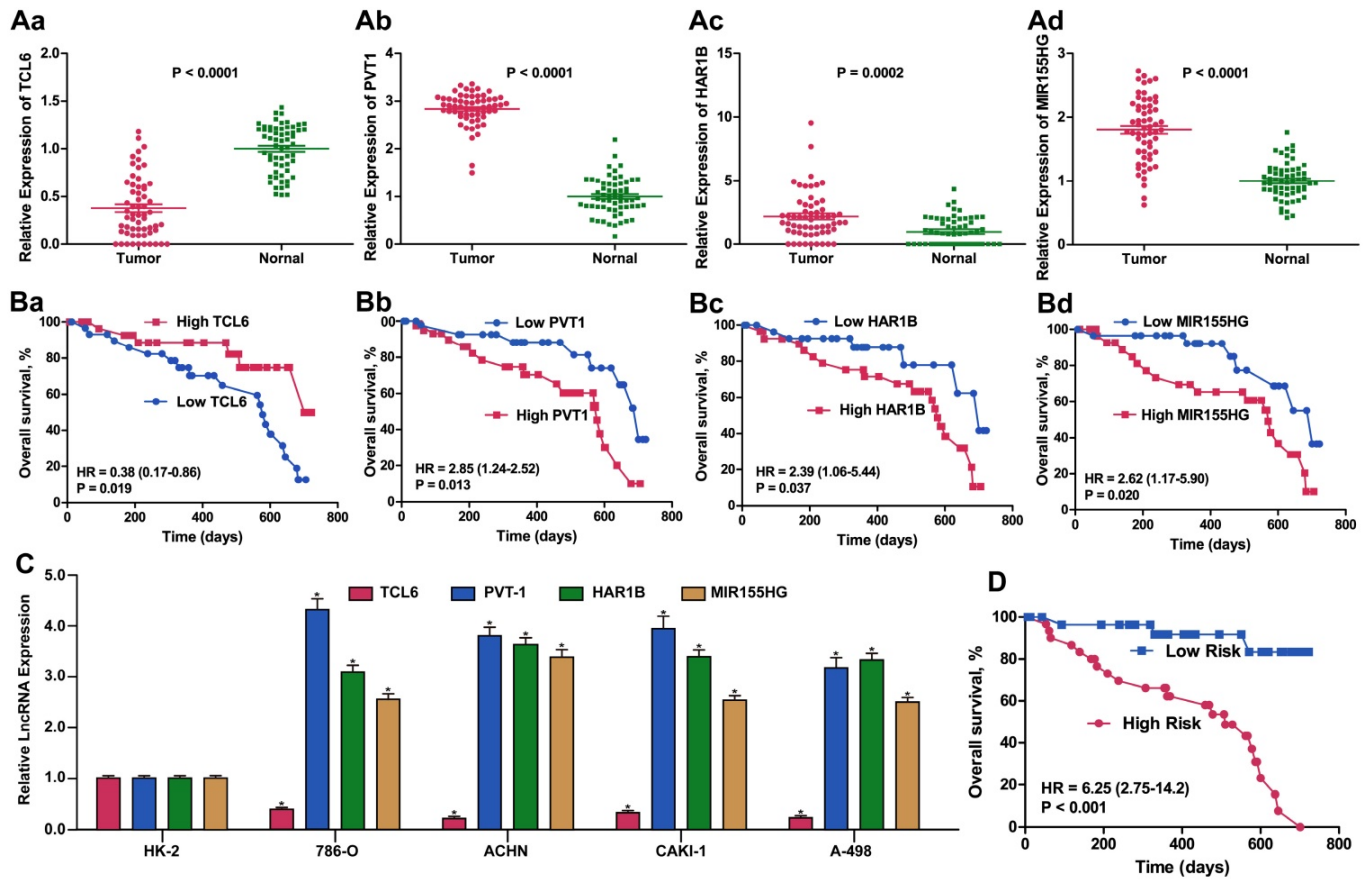
** Expression and Kaplan-Meier OS analysis of the 4-lncRNA signature in our validation cohort of ccRCC patients.** Relative fold change of validated expression of the four lncRNAs in the Tongji ccRCC cohort compared with normal adjacent tissue determined by qPCR. (B). Kaplan-Meier survival analysis of the four lncRNAs in the Tongji ccRCC validation cohort. (C) Relative fold change of expression of the four lncRNAs in 786-O, ACHN, CAKI-1 and A498 ccRCC cell lines compared with the immortalized proximal tubule epithelial cell line HK2, as determined by qPCR. (D). Kaplan-Meier survival analysis of the 4-lncRNA signature in the Tongji ccRCC validation cohort.

Considering the basis of the lncRNA profiling outcomes in TCGA, we further detected ccRCC-related lncRNA expression using qPCR to examine 60 ccRCC samples from Tongji Hospital to estimate and validate the value of the candidate lncRNAs for prognosis. We selected the panel of four lncRNAs for this qPCR verification analysis. The expression level of TCL6 was decreased whereas the levels of PVT1, MIR155HG and HAR1B were increased in ccRCC tissues compared with adjacent normal tissue (all p < 0.01). Similar results for expression of these four lncRNA were obtained when detected in four RCC cell lines compared with HK2 cells. The same prognostic score formula acquired from the dataset from TCGA was utilized to calculate the four-lncRNA signature score for each of the 60 patients in our validation cohort. Using the median value as the optimum cut-off point, we validated the four-signature lncRNAs as a potential prognostic biomarker (HR: 6.25, 95%CI: 2.75-14.2, p < 0.001) (Figure 5).

## Discussion

Many lncRNAs with abnormal expression that is highly related to different cancer types have been identified through genome-wide transcriptome analyses 8. A series of studies have revealed that lncRNAs can act as regulators of diverse biological functions, including X-chromosome silencing, transcription regulation, and genomic stability 12, 13. Recently, some studies have evaluated the prognostic relevance of lncRNAs in ccRCC patients, though most have focused on limited lncRNAs with a small number of patients. Therefore, lncRNA signatures might have concrete predictive and prognostic value in the management of RCC. The purpose of our study was to identify a lncRNA signature using TCGA data that is able to predict prognosis in RCC. A total of 525 RCC patients with corresponding clinical data were enrolled, and lncRNAs significantly related to overall survival (OS) in RCC patients were assessed in a Cox proportional regression model. We generated a risk-score formula to examine the value of the lncRNA signature in predicting RCC prognosis. Four lncRNAs were confirmed to be markedly related to OS in RCC patients. Patients with high risk scores experienced lower overall survival than patients who had low risk scores, and multivariate Cox regression analyses showed that the lncRNA signature could perform independently as an indicator of prognosis. In addition, the signature was validated as a predicator in our Tongji ccRCC cohort. In summary, our study identified a 4-lncRNA signature that could serve as an independent marker in the prognosis of ccRCC.

Compared with previous studies, our study utilized data from TCGA with high throughput analysis of lncRNAs. A total of 1056 lncRNAs were initially included in the present study, offering a more comprehensive analysis. Furthermore, the significance level was set as 0.001 and the fold change as 1.5 to control the false discovery rate. By combining the 4 identified lncRNAs, the lncRNA signature risk score may act as an independent predictor in ccRCC.

During the training phase, we probed the expression profiles of 60 candidate lncRNAs in ccRCC tissues and adjacent normal kidney tissues; among these, four (TCL6, PVT1, MIR155HG and HAR1B) showed evidently disparate expression between the tissues. TCL6 expression was notably reduced in ccRCC tissues, whereas expression of PVT1, MIR155HG and HAR1B was increased, and this trend was in accordance with previous studies 18, 19.

The T-cell leukemia/lymphoma 6 (TCL6) locus maps 7 kb centromeric to the TML1 locus and is composed of at least 12 exons with small alternative exons 20. Similar to our results, Su et al. reported that TCL6 was overexpressed in RCC; decreased TCL6 expression suggested an inferior prognosis for patients with ccRCC. Furthermore, overexpression of TCL6 in 786-O and Caki-1 RCC cells decreased proliferation and increased apoptosis compared to controls 19.

PVT1 is a widely reported oncogene that may be involved in renal cancer, lung cancer, colorectal cancer, gastric cancer, hepatocellular carcinoma, ovarian cancer, and leukaemia 21-24. Among all cancer types, renal clear cell carcinoma displays the strongest upregulation of PVT1, and its misregulation in ccRCC is largely associated with promoter hypomethylation 18. Indeed, Wang et al. reported that PVT1 overexpression in hepatocellular carcinoma cells enhanced cell proliferation, cell cycling, and the acquisition of stem cell-like properties by stabilizing NOP2, suggesting that it may act as an oncogene in ccRCC progression 25. Xu et al. also reported that high expression of PVT1 predicted an inferior prognosis in patients with gastric cancer. By interacting with FOXM, prominent expression of PVT1 enhanced cancer proliferation and invasion 24. A serum 5-lncRNA signature that includes PVT1 was recently discovered as a biomarker facilitating the detection of ccRCC 26. Consistent with our results, Posa et al. found PVT1 to serve as a prognostic factor for novel therapeutic interventions in RCC 18.

The miR-155 host gene (MIR155HG), which is located on chromosome 21, encodes miR-155 27; miR-155 expression has been confirmed to be up-regulated in ccRCC tissue and cell lines, and it may function as an oncogene by targeting BACH1 28, 29. Recent studies have provided evidence that MIR155HG mainly influences B-cell receptor signaling and is associated with aggressive disease in leukaemia 30. Furthermore, Wang et al. reported that MIR155HG, as one of a 4-lncRNA signature, had prognostic value for anaplastic glioma and was increased with tumor grade 31. We found both HAR1A and HAR1B to be up-regulated in ccRCC and associated with poor survival. Similar to our results, a 9-lncRNA signature consisting of HAR1A and its upregulation predicted breast cancer recurrence and served as a prognostic marker for breast cancer 32. Using TCGA, Ma et al. investigated lncRNAs as prognostic biomarkers for papillary thyroid cancer, revealing that low HAR1A expression was associated with cancer recurrence and poor prognosis 33. Regardless, there have been no reports on MIR155HG and HAR1B in ccRCC to date. Therefore, exploring their roles in tumorigenesis may contribute to demonstrating their oncogenic or suppressor function in ccRCC patients.

No comprehensive analysis to investigate lncRNA profiling in RCC has been performed thus far. However, the results of our study are helpful for exploration of potential lncRNA biomarkers in human ccRCC. We suggest four promising lncRNAs that have been abundantly reported as having altered expression and significant dysregulation. Nonetheless, this panel is only specific for ccRCC. Furthermore, some limitations existed in our study that should be considered. First, it should be noted that a large portion of unknown lncRNAs were missing due to the intrinsic limitation of the microarray technique and probe repurposing method. Second, only the dataset (KIRC) from TCGA and our ccRCC cohort were evaluated in this research, resulting in inadequate samples for the 4-lncRNA signature model of prognosis. Accordingly, further studies with larger cohorts are warranted to validate our prognostic model. Finally, ccRCC covers most of RCC cases. When validating our prognostic model in kidney renal papillary cell carcinoma (KIRP) and kidney chromophobe carcinoma (KIRC), the results showed no significant difference. Thus, we suggest that the significant prognostic value of the lncRNA signature be further assessed in other subtypes of RCC.

## Conclusions

In summary, by analyzing the genome-wide lncRNA expression profiles from TCGA, a panel of four lncRNAs was identified that may serve as an independent predictor of prognosis in ccRCC. With further confirmation of the mechanisms by which these lncRNAs impact ccRCC progression, the 4-lncRNA signature might not only have prognostic value for low-risk patients who will benefit from nephroectomy but may also provide a deeper understanding of the molecular heterogeneity of ccRCC. Therefore, more research is needed to uncover novel diagnostic or prognostic lncRNA candidates and elucidate their function in ccRCC.

## Supplementary Material

Supplementary figures and tables.

## Figures and Tables

**Table 1:** Univariate and multivariate Cox proportional hazards analyses of overall survival for patients with ccRCC in TCGA.

Variables	Categories	Univariate Analysis			Multivariate Analysis		
		HR	95% CI	P Value	HR	95% CI	P Value
Age	< 65 vs. ≥ 65 ys	1.014	1.024-1.058	**<0.001**	1.031	1.015-1.047	**<0.001**
Sex	Male vs. female	0.863	0.589-1.266	0.452	0.976	0.691-1.381	0.892
Laterality	Left vs. Right	1.618	1.117-2.343	**0.011**	1.185	0.851-1.649	0.315
Dimension	< 1.5 vs. ≥ 1.5 cm	1.730	1.296-2.310	**<0.001**	0.842	0.659-1.076	0.17
tumor stage	T4vs.T3vs.T2vs.T1	2.377	1.986-2.846	**<0.001**	1.564	1.238-1.977	**<0.001**
tumor grade	G4vs.G3vs.G2vs.G1	1.322	0.897-1.365	0.236	1.127	0.907-1.4	0.279
Lymph node	Negative vs. positive	0.805	0.667-0.971	**0.024**	0.841	0.707-1	0.05
Metastasis	Yes vs. No	2.231	1.579-3.152	**<0.001**	1.636	0.957-2.797	0.072
TCL6	High vs. Low	0.484	0.357-0.658	**<0.001**	0.947	0.878-1.023	0.166
MIR155HG	High vs. Low	1.763	1.298-2.394	**0.0003**	1.065	0.949-1.195	0.282
HAR1B	High vs. Low	1.564	1.143-2.129	**0.0051**	1.211	1.016-1.443	**0.032**
PVT1	High vs. Low	1.791	1.318-2.434	**0.0002**	1.155	0.938-1.422	0.175
Risk Score	High vs. Low	2.57	1.89-3.5	**<0.001**	1.932	1.392-2.682	**<0.001**
