# Supplementary material for: A Panel of Four-lncRNA Signature as a Potential Biomarker for Predicting Survival in Clear Cell Renal Cell Carcinoma
Source: J Cancer. 2020 Apr 27;11(14):4274–83. doi: 10.7150/jca.40421 (PMC7196268; doi:10.7150/jca.40421)
Supplement: Supplementary file 1 — Supplementary figures and tables. [file jcav11p4274s1.pdf]

Electronic Supplementary Figures

Figure Legends

Figure S1. Kaplan-Meier overall survival analysis of lncRNAs using the KIRC TCGA dataset.

The optimal cut-off point for lncRNA expression determined by the median value was applied to the validation cohort and reached high statistical significance. The plot shows the chi-squared log-rank values obtained when the cohort was divided into two groups.

Figure S2. Relative lncRNA expression by risk group in ccRCC patients from TCGA.

Figure S3. Kaplan-Meier overall survival analysis of the 4-lncRNA signature subgrouped by class grade using the KIRC TCGA dataset.

A, Overall survival analysis subgrouped by grade using the overall KIRC TCGA dataset. B, Overall survival analysis of the 4-lncRNA signature using the grade 2 KIRC TCGA dataset. C, Overall survival analysis of the 4-lncRNA signature using the grade 3 KIRC TCGA dataset. D, Overall survival analysis of the 4-lncRNA signature using the grade 4 KIRC TCGA dataset.

Figure S4. Kaplan-Meier overall survival analysis of the 4-lncRNA signature subgrouped by class/stage using the KIRC TCGA dataset.

A, Overall survival analysis subgrouped by stage using the overall KIRC TCGA dataset. B, Overall survival analysis of the 4-lncRNA signature using the stage II KIRC TCGA dataset. C, Overall survival analysis of the 4-lncRNA signature using the stage III KIRC TCGA dataset. D, Overall survival analysis of the 4-lncRNA signature using the stage IV KIRC TCGA dataset.

Figure S5. The predictive value of the 4-lncRNA signature in the KIRP (A) and KICH (B) TCGA datasets.

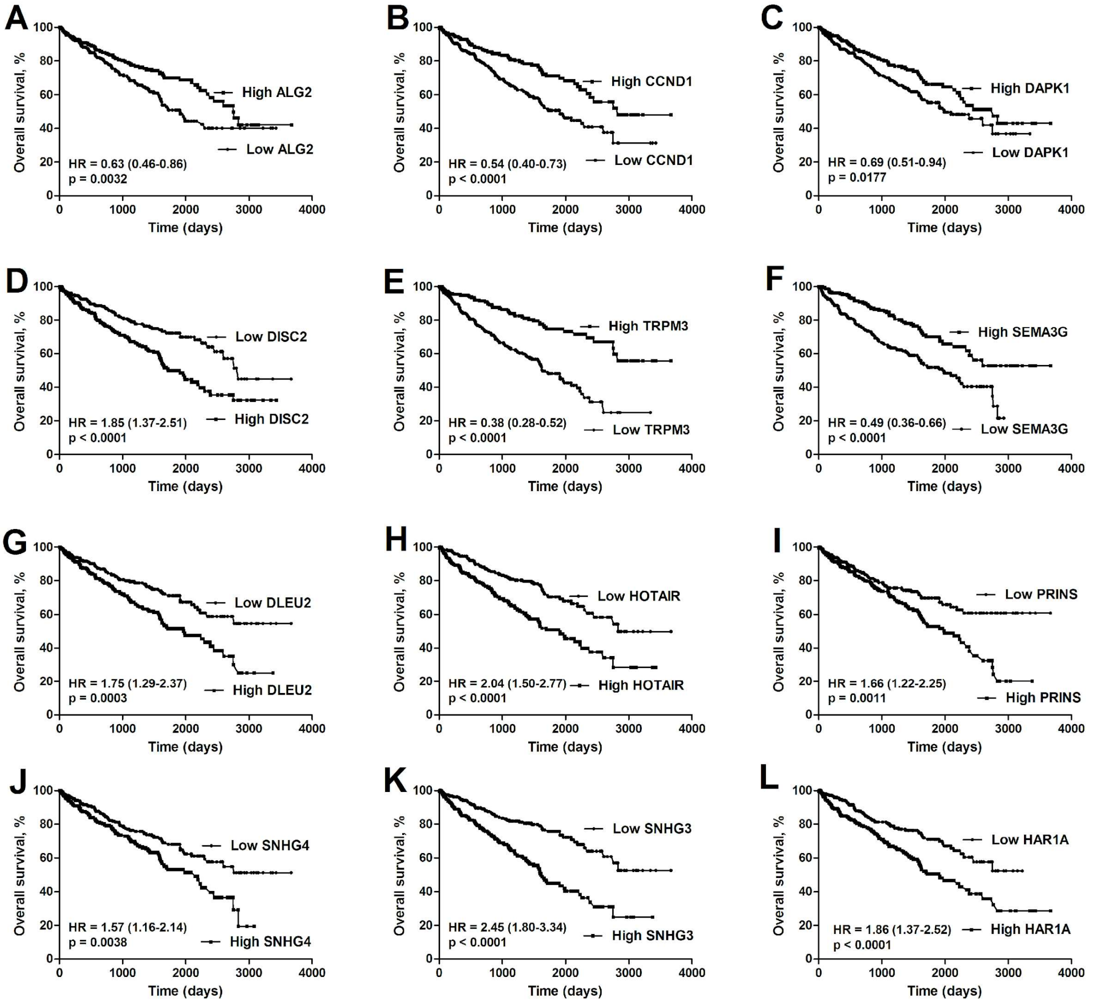

Figure S1. Kaplan-Meier overall survival analysis of lncRNAs using the KIRC TCGA dataset.

The optimal cut-off point for lncRNA expression determined by the median value was applied to the validation cohort and reached high statistical significance. The plot shows the chi-squared log-rank values obtained when the cohort was divided into two groups.

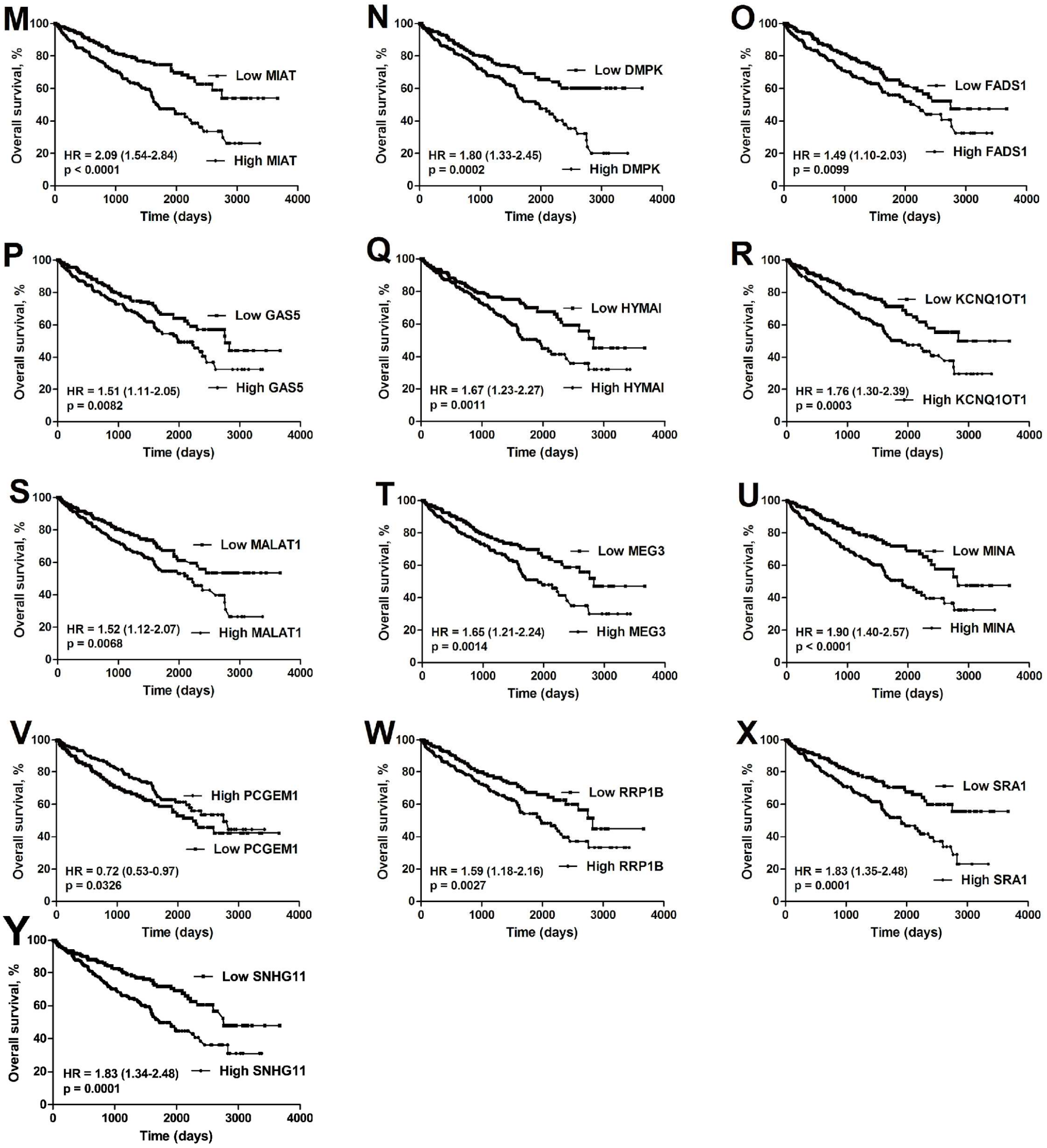

Figure S1 (Continued)

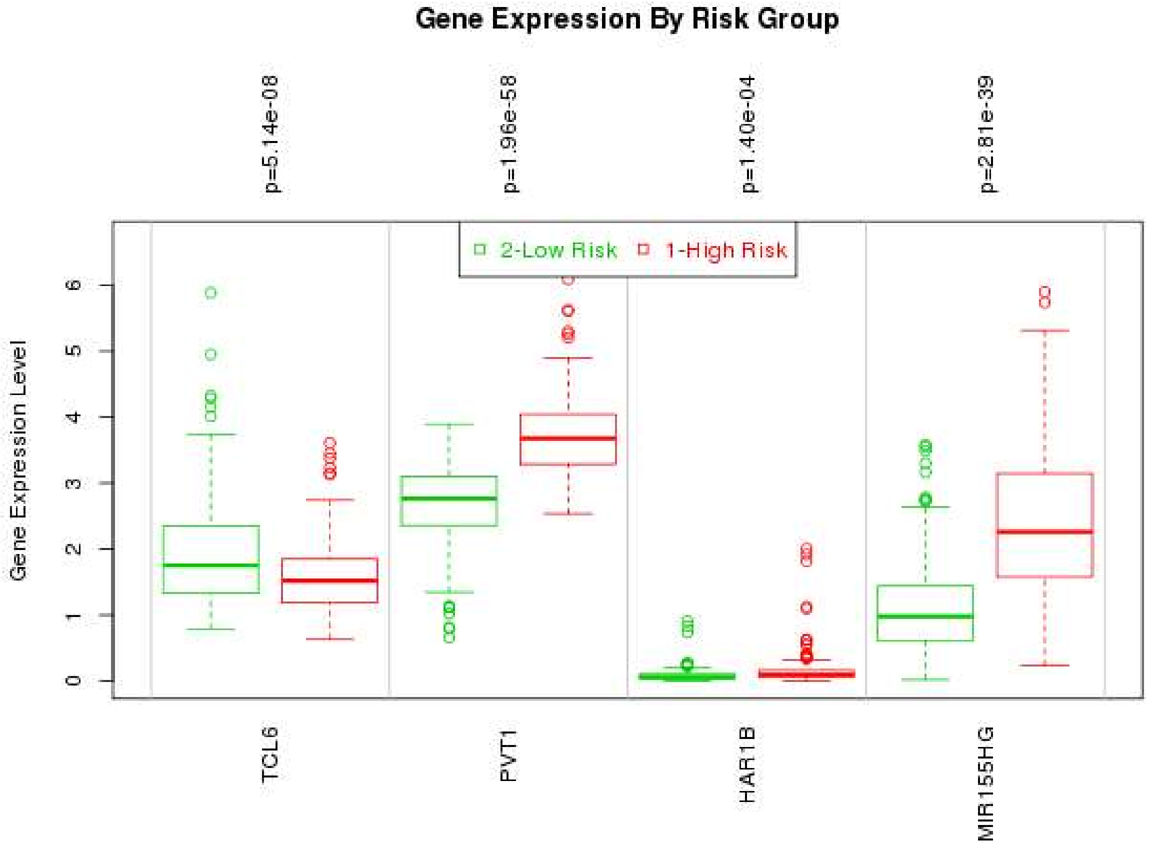

Figure S2. Relative lncRNA expression by risk group in ccRCC patients from TCGA.

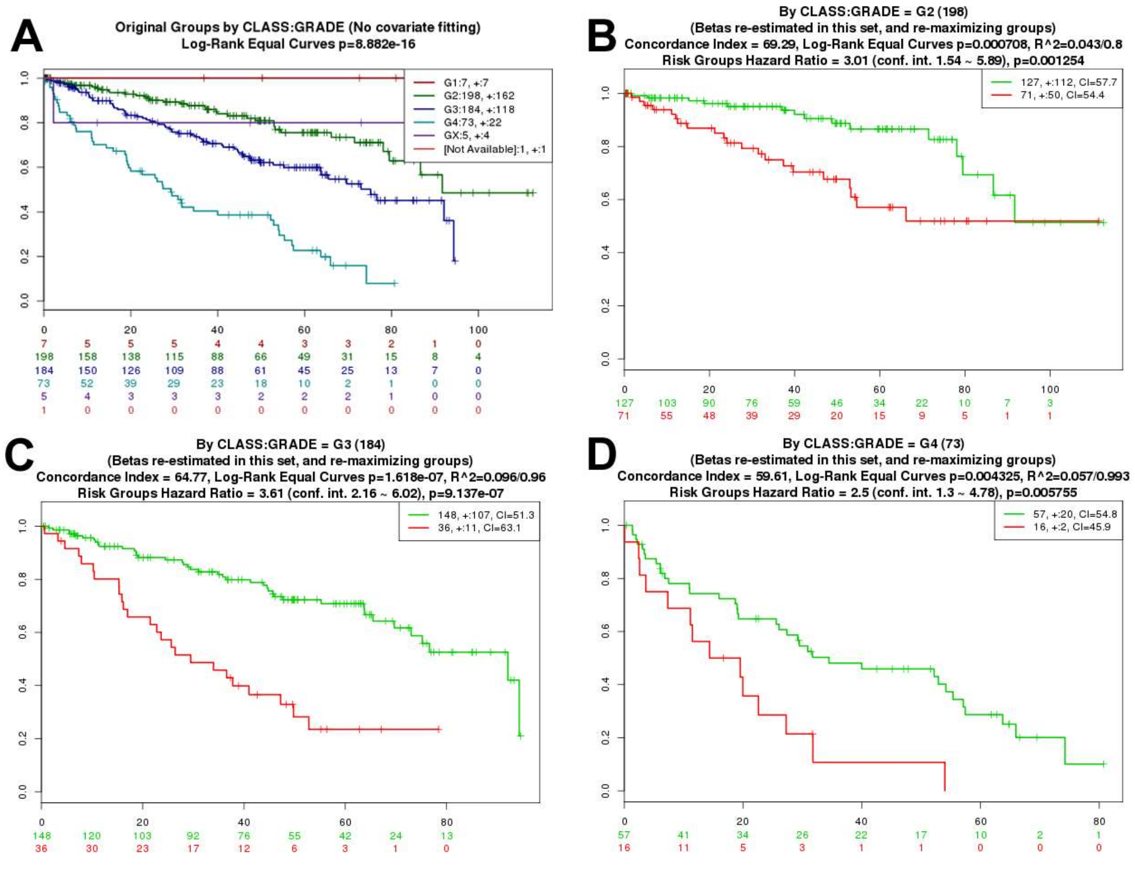

Figure S3. Kaplan-Meier overall survival analysis of the 4-lncRNA signature subgrouped by class/grade using the KIRC TCGA dataset.

A, Overall survival analysis subgrouped by grade using the overall KIRC TCGA dataset. B, Overall survival analysis of the 4-lncRNA signature using the grade 2 KIRC TCGA dataset. C, Overall survival analysis of the 4-lncRNA signature using the grade 3 KIRC TCGA dataset. D, Overall survival analysis of the 4-lncRNA signature using the grade 4 KIRC TCGA dataset.

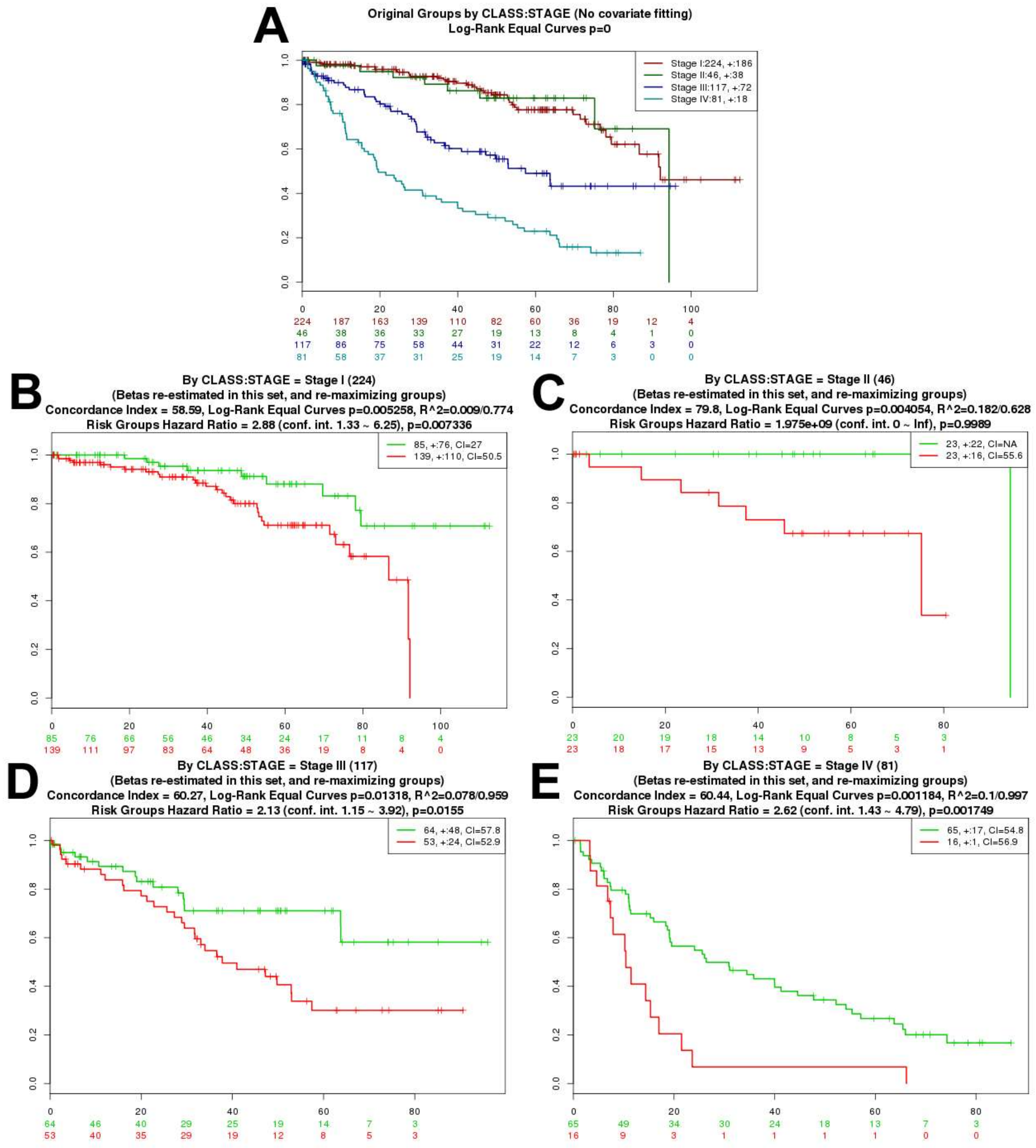

Figure S4. Kaplan-Meier overall survival analysis of the 4-lncRNA signature subgrouped by class/stage using the KIRC TCGA dataset.

A, Overall survival analysis subgrouped by stage using the overall KIRC TCGA dataset. B, Overall survival analysis of the 4-lncRNA signature using the stage II KIRC TCGA dataset. C, Overall survival analysis of the 4-lncRNA signature using the stage III KIRC TCGA dataset. D, Overall survival

analysis of the 4-lncRNA signature using the stage IV KIRC TCGA dataset.

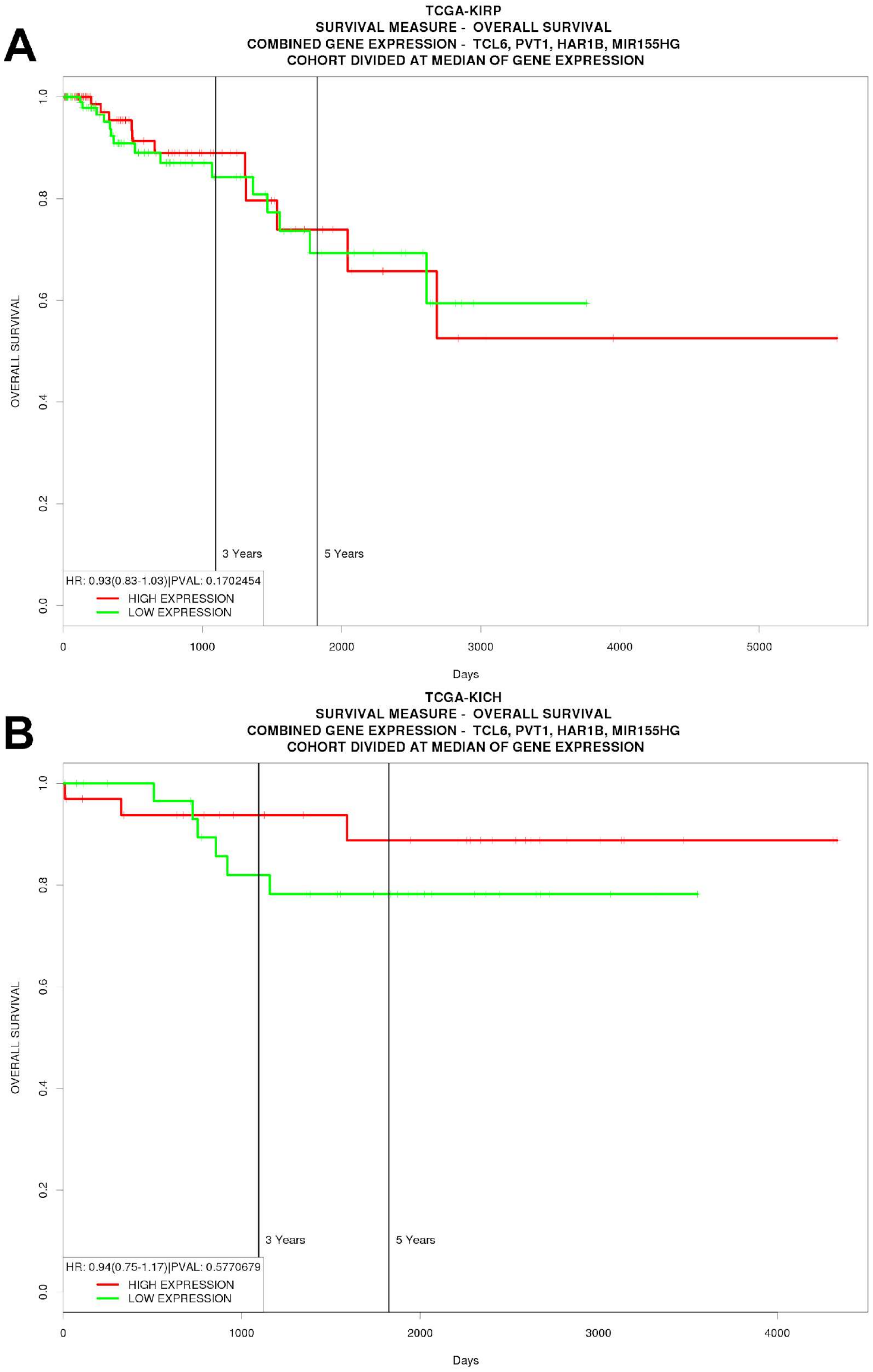

Figure S5. The predictive value of the 4-lncRNA signature in the KIRP (A) and KICH (B) TCGA datasets.

Electronic Supplementary Tables

Table S1. Clinical characteristics of patients with ccRCC in TCGA and the Tongji cohort.

**Variable**	**TCGA cohort (N=525)**		**Tongji cohort (N=60)**	
	**N**	**%**	**N**	**%**
**Age, median (range) year**	61 (26-90)		58 (47-79)	
**Gender**				
Male	341	65.0	43	71.7
Female	184	35.0	17	28.3
**Grade**				
G1 & G2	240	45.7	29	48.3
G3 & G4	277	52.8	31	51.7
Gx	8	1.5	0	0.0
**Dimension, mean (range) cm**	1.69 ±0.66		4.98 ± 1.85	
**pT**				
T1	267	50.9	42	70.0
T2	68	13.0	9	15.0
T3	179	34.1	7	11.7
T4	11	2.1	2	3.3
**N**				
N0	271	51.6	55	91.7
N1	17	3.2	2	3.3
Nx	237	45.1	3	5
**M**				
M0	421	80.2	53	88.3
M1	79	15.0	3	5.0
Mx	25	4.8	4	6.7
**Stage**				
I	262	49.9	43	71.7
II	56	10.7	7	11.7
III	126	24.0	6	10.0
IV	81	15.4	4	6.7
**Position**				
Left	247	47.0	28	46.7
Right	277	52.8	29	48.3
Bilateral	1	0.2	3	5.0

Table S2. Top significantly dysregulated lncRNAs in clear renal cell carcinoma tissue compared with normal adjacent tissue using the KIRC TCGA dataset.

lncRNAs	FC	p value
**Downregulated lncRNAs**		
UCA1	0.27	3.42332E-27
C15orf2	0.29	8.12677E-54
LOC728606	0.45	3.66335E-32
TCL6	0.51	3.07675E-26
LOC554202	0.55	9.95879E-18
TERC	0.73	0.037004565
TRPM3	0.74	2.09929E-15
ASFMR1	0.77	0.006229033
RMST	0.78	0.010743939
SEMA3G	0.81	1.60935E-27
CASC2	0.83	1.86509E-36
**Upregulated lncRNAs**		
MIR17HG	1.20	0.000600988
DLEU2	1.28	1.09096E-35
HOTAIR	1.31	4.88856E-08
PRINS	1.37	0.00010277
SNHG3	1.40	1.18776E-19
SNHG4	1.42	1.77625E-07
HAR1A	1.44	7.84311E-11
MIAT	1.48	1.95045E-26
XIST	1.64	0.000175075
MIR155HG	1.98	2.23414E-39
DGCR5	2.14	7.91298E-81
HAR1B	2.17	1.74739E-07
PSORS1C3	2.28	1.9153E-28
PTHLH	2.52	1.87169E-37
PVT1	2.96	1.5916E-148

Table S3. List of lncRNA primers used for qRT-PCR validation in this study.

lncRNA	(5'-3')	SEQUENCE (5'-3')
Tcl6	Forward Primer	TCGGACGAAGTGTTTCCAGACC
	Reverse Primer	TGCAGAGGCTCTCAAGCTGTAG
PVT1	Forward Primer	TTACAGGCGTGTGCCACAAAGC
	Reverse Primer	GCCTGTAATCCCAGCACGTTGA
MIR155HG	Forward Primer	AGGGGTTTTTGCCTCCAACT
	Reverse Primer	TCTTTGTCATCCTCCCACGG
HAR1B	Forward Primer	CGTCTCCTCCGTTTCATGCT
	Reverse Primer	ATCCCTGAATGTCCACACCG

Table S4. Overall information on the four ccRCC-specific lncRNAs.

lncRNA	Description	Ensembl	Synonyms	Chromosome
TCL6	T-cell leukaemia/lymphoma 6	ENSG00000187621	TNG1, TNG2	14q32.13
PVT1	Pvt1 oncogene	ENSG00000249859	NCRNA00079, LINC00079, MYC, onco-lncRNA-100	8q24.21
MIR155HG	MIR155 host gene	ENSG00000234883	BIC, NCRNA00172	21q21.3
HAR1B	highly accelerated region 1B	ENSG00000231133	HAR1R, NCRNA00065, LINC00065	20q13.33
